# The Copepoda mitogenome as a dynamic evolutionary landscape

**DOI:** 10.1371/journal.pone.0350115

**Published:** 2026-06-10

**Authors:** Javier Urban-Olivares, Elizabeth Ortega-Mayagoitia, José Arturo Alcántara-Rodríguez, Nohemi Dimas-Flores, Alejandra Rougon-Cardoso, Jorge Ciros-Pérez

**Affiliations:** 1 Posgrado en Ciencias Biológicas, Universidad Nacional Autónoma de México. Cto. de Posgrados, Ciudad Universitaria, Coyoacán, Ciudad de México, Mexico; 2 Facultad de Estudios Superiores Iztacala, Laboratory of Evolutionary Biology of Plankton, Tropical Limnology Group – UIICSE, División de Investigación y Posgrado, Universidad Nacional Autónoma de México, Tlalnepantla de Baz, Edo. de México, Mexico; 3 Laboratory of Agrigenomic Sciences, Universidad Nacional Autónoma de México, ENES-León, León, Mexico; Central University of South Bihar, INDIA

## Abstract

Copepods are an extraordinarily diverse group that exhibit a broad spectrum of morphological, physiological, life-history traits, and habitat specializations. Despite their ecological, evolutionary, and economic importance, molecular resources are scarce, limiting our understanding of their diversification and adaptation. We analysed the evolution of copepod mitogenomes at different phylogenetic scales using 19 complete mitogenomes deposited in GenBank along with five *de novo* assemblies from species of the genus *Leptodiaptomus* from Central Mexico. All five new mitogenomes are circularized and include all canonical metazoan coding regions but differ in the composition and size of non-coding regions (NCRs). The mitochondrial genomes of the four populations of the *L. sicilis*-group are the largest reported to date in Copepoda (>36,000 bp). The NCRs of *Leptodiaptomus* spp. contain repeated regions, pseudogenes, long palindromes with secondary structures, and open reading frames, although much of their content is still unexplained. Gene ordering in Copepoda is highly dynamic, and even gene blocks highly conserved across metazoans are either absent or occur at a low frequency. In Calanoida, the NCRs have expanded considerably, whereas in podoplean clades (Cyclopoida, Harpacticoida, and Siphonostomatoida), they remain similar to the hypothetical ancestral state. While some genes display evident signatures of purifying selection, most exhibit evidence of positive selection across all branches of the phylogeny. These findings offer a basis for further research on the molecular mechanisms driving copepod adaptation and diversification, as well as for refining predictions of their responses to environmental change.

## Introduction

Mitochondria are essential organelles present in almost all eukaryotic cells [1]. One of their primary functions is oxidative phosphorylation, a process in which the respiratory chain, which is embedded within the cristae membrane, recycles adenosine diphosphate (ADP) into adenosine triphosphate (ATP), the main energy currency of the cell [2,3]. Additionally, mitochondria play broader roles in critical cellular processes such as apoptosis, aging, intracellular signalling, metabolic homeostasis, and macromolecule biosynthesis [4]. The advent of sequencing and bioinformatics technologies has expanded our capacity to explore adaptive evolution at the mitochondrial level [5], leading to the discovery of diverse structural and functional features [6].

The mitogenomes of eumetazoans are typically small, usually ranging between 14,000 and 20,000 base pairs, although considerably larger mitogenomes have occasionally been reported [7–10]. They consist of a double-stranded circular DNA molecule containing 37 genes: 13 protein-coding genes (PCGs) that encode subunits of respiratory chain complexes I (*ND1*–*ND6* and *ND4L*), III (*CYTB*), IV (*COX1*–*COX3*), and V (*ATP6* and *ATP8*); 22 genes encoding transfer RNAs (tRNAs); and two ribosomal RNA genes (*12S rRNA* and *16S rRNA*). An increasing number of mitogenomes deviate from this canonical structure, especially in invertebrates, which exhibit significant variation in genome size [7,11] and distinct modes and rates of molecular evolution. Tandem Duplication–Random Loss (TDRL) events have shaped gene order and genome size [12]. Variations in mitogenome size are largely attributed to differences in the length and organization of non-coding regions or NCRs, which are rich in A + T and may include tandem repeats or stem‒loop motifs (reviewed previously [13]). Some of these repeated elements are considered mobile and may play roles in recombination, replication initiation, and transcription [8,14].

Copepods represent a highly diverse group, exhibiting variability in morphology, physiology, life-history traits, and habitat occupancy. Their origin is estimated to date back to the early Palaeozoic, with the divergence of the two main superorders, Podoplea and Gymnoplea, occurring between the late Cambrian and Devonian periods (~446.2 ± 47.3 Mya) [15,16]. Today, more than 14,800 species are recognized [17] and are among the most abundant groups of multicellular organisms in all aquatic environments. They inhabit a wide range of habitats, including groundwater, caves, ponds, streams, lakes, sediments, oceans, and even leaf litter. Ecologically, they serve as key components in food webs and as sensitive indicators of environmental change; economically, some species are parasites or predators of aquaculture species and potential vectors of waterborne diseases [17,18]. Despite their importance, mitochondrial genomic studies in copepods remain limited, and further research is needed to understand how mitochondrial evolutionary processes contribute to their diversification and broad ecological adaptability. Previous studies have shown that copepod mitogenomes exhibit substantial size variation (14,000–28,000 bp), primarily due to expanded NCRs [19,20] and unusual gene structures and arrangements [10,21]. The latter pattern contrasts with that of other crustaceans, such as Branchiopoda, which also originated in the middle Cambrian (478–512 Mya; [22]) and has diversified across marine and inland waters but relatively conserved mitochondrial gene arrangements [23,24].

In this study, we analysed and compared complete mitochondrial genomes of Copepoda to describe evolutionary patterns of mitochondrial genome architecture across this group. Our dataset included 19 previously published and publicly available complete mitogenomes from the NCBI GenBank database, as well as five *de novo* assembled mitogenomes from populations of the genus *Leptodiaptomus* inhabiting ecologically distinct but geographically proximate lakes in Central Mexico [25,26]. One of these populations corresponds to the species *L. garciai* [27], which is endemic to Lake Alchichica [28,29]. The other four represent closely related lineages within the *L. sicilis* group [30], which share morphological and phylogenetic affinity with *L. sicilis* [31] *sensu stricto*. These lineages are adapted to divergent ecological niches and form monophyletic groups with independent evolutionary trajectories [30,32]. Using the complete 24 genomes, we explored mitochondrial genome evolution during copepod diversification, examining multiple phylogenetic scales, from ancient divergences, such as the split between Gymnoplea (Calanoida) and Podoplea (Cyclopoida, Harpacticoida, and Siphonostomatoida), to the recent divergence within the *L. sicilis*-group. Our overarching goal is to provide a novel perspective on mitochondrial genome evolution in copepods and to offer a foundation for future studies that link mitogenomic dynamics with adaptive evolution in this ecologically successful group.

## Materials and methods

### Sampling and DNA extraction

The populations of *Leptodiaptomus* spp. inhabit five different lakes located in the Cuenca Oriental in Central Mexico. *L. garciai* is a microendemic species of Lake Alchichica, and the four populations of the *L. sicilis* group inhabit the lakes Atexcac, El Carmen, La Preciosa and Quechulac (Table 1). The samples were collected at the center of each lake with vertical tows in June 2020 and February 2022 using a conical plankton net with a mesh size of 80 μm. The samples were kept alive and analysed in the laboratory to identify each copepod specimen using standard procedures in the taxonomy of the *Leptodiaptomus* genus [33]; approximately 200 adult individuals were obtained from each population. The copepods were subsequently fasted for 48 hours in a medium prepared to match the salinity of their respective lake (Table 1) using commercial salts (Instant Ocean®) and deionized water (Millipore®, Elix 5). A 5% solution of penicillin was added to minimize the potential contribution of the intestinal contents and/or bacteria during genomic material extraction. Owing to the small size of adult copepods (0.75–1.39 mm long, average individual biomass ≈ 6.5 μg [32]), each DNA sample was obtained by pooling the set of 200 individuals from each population. DNA extraction was performed using the DNeasy Blood and Tissue Kit (Qiagen, Valencia, CA, USA). Total DNA was quantified by fluorometry with a QubitTM 3 fluorometer to achieve a concentration >95 ng/μL, and a 1% agarose gel was run to check DNA integrity and to avoid fragments <10 kb.

**Table 1:** Key ecological features and geographic location of the lakes inhabited by *L. garciai* (Alchichica) and the *Leptodiaptomus sicilis-*group populations (Atexcac, La Preciosa, Quechulac and El Carmen).

	Lake Alchichica	Lake Atexcac	Lake La Preciosa	Lake Quechulac	Lake El Carmen
Location	19° 24’ N, 98° 24’ W	19°200 N, 97°270 W	19°220 N, 97°230 W	19°22’ N, 97°21’ W	19°26’ N, 97°47’ W
Lake type	Maar	Maar	Maar	Maar	Playa
Permanence	Perennial	Perennial	Perennial	Perennial	Ephemeral
Area (km^2^)	1.9	0.2	0.7	0.5	~290^a^
Z_max_ (m)	65	35	45	35	0-0.30
pH	8.7	8.4	8.6	8.7	8.7-11.2
Salinity (TDS, g/L)	9.0	6.5	1.1	0.4	1.6-48.0
Fish presence	Yes	No	Yes	Yes	No

Abbreviations: TDS, total dissolved solids without temperature normalization; *Z*_max_, maximum depth. ^a^ Inundation area.

### Sequencing, assembly and annotation

Genomic DNA samples that passed quality testing were sent to the Genomics Center at the University of Minnesota (https://genomics.umn.edu/) for whole-genome sequencing. Four TruSeq Nano, Unique Dual-Indexed (UDI) DNA libraries were created using Illumina™ technology; all the libraries were pooled for single-lane sequencing of a 2 × 150 bp NovaSeq 6000 System Prime flow cell, yielding >375 M reads with average quality scores ≥ Q30 for all the libraries.

Long-read genomic DNA sequences (PacBio > 50 kb) were also obtained from the *L. sicilis*-group from the Atexcac population by creating a 20 kb PacBio Express library at the University of Minnesota Genomics Center (UMGC). Sequencing was performed on a Sequel II 8 M SMRT cell using the contiguous long-read (CLR) method, which generated 108 Gb of sequences and 48 Gb of unique sequences for the sample, with an N50 read size of ~15 kb and an average of 10 kb.

Given that our sequences originated from whole-genome sequencing, mitochondrial sequences were identified by alignment with 19 published complete and circularized copepod mitogenomes to optimally recover all coding and non-coding regions using BWA v0.7 [34] and SAMtools v0.1.20 [35] (S1 Table in S1 File). The raw mitochondrial sequences (Table 2) were assembled *de novo* using Unicycler v0.4.9 [36]. A hybrid assembly was constructed for the *L. sicilis*-group Atexcac mitogenome, for which long-read data were available, allowing for a more accurate and contiguous mitogenome assembly. The *L*. *sicilis*-group mitogenomes (Carmen, Preciosa and Quechulac) were assembled by remapping their reads to a manually curated version of the *L. sicilis*-group Atexcac mitogenome. This curation aimed to correct minor inconsistencies in regions with low coverage. The resulting mapped reads were carefully examined to confirm consistent coverage and the absence of discrepancies in the final assemblies. The BAM files were used to assess and visualize the coverage of each *de novo* mitogenome using Geneious ^(R)^ v9.1.8 [37].

**Table 2:** Overview of the size and coverage of the *de novo* mitochondrial assemblages of *Leptodiaptomus* spp. copepods. The four populations of the *L. sicilis*-group are named according to the lakes they inhabit. NCR = Non-coding region.

Species	Genome size (bp)	NCR size (bp)	Assembled Short Reads	Assembled Long Reads	% A + T	NCBI Accession Number of mtDNA	NCBI Accession Number of Reads
*L. garciai*	14,655	609	303,442	–	68.2	PQ586096	SRR31168557
*L. sicilis*-group							
Atexcac	36,727	22,549	288,120	18,306	70.9	PQ586097	SRR31168556
Carmen	36,745	22,625	464,272	–	70.9	PQ586098	SRR31168555
Preciosa	36,642	22,585	440,022	–	71.0	PQ586099	SRR31168554
Quechulac	36,685	22,568	458,964	–	70.9	PQ586100	SRR31168553

The functional annotations of the assembled mitogenomes were performed using MitoZ v2.4 [38], the invertebrate mitochondrial genetic code and the arthropod database. Initially, tRNAscan-SE v2.0 [39] was employed but failed to detect all the tRNAs. A subset of eight tRNAs was subsequently identified searching for sequence similarity against other copepod genomes using Geneious^(R)^ v9.1.8. To validate the 22 annotated tRNAs, the predicted secondary structure folding was performed using the Vienna Package v2.5.1 [40]. Using a copepod mitogenome database, manual curation was performed to refine and correct functional genetic annotations with Geneious^(R)^ v9.1.8 and BLAST for confirmation.

The nucleotide composition of the mitogenomes was analysed with MEGA-X [41]. The relative synonymous codon usage (RSCU) of the 13 PCGs was determined using the CAIcal server [42] and subsequently plotted with ggplot2 in R [43,44].

### Identification and analysis of non-coding regions

Patterns of repeated sequences within the non-coding regions (NCRs) of the five mitochondrial genomes of *Leptodiaptomus* copepods were searched and analysed using the Tandem Repeats Finder web server (https://tandem.bu.edu/trf/trf.html) [45]. In addition, palindromic regions were identified using Geneious^(R)^ v9.1.8, and the resulting sequences were folded with Vienna v2.5.1 (S7 Fig in S1 File). Open reading frames (ORFs) were identified using Geneious^(R)^ v9.1.8, and a BLAST analysis was performed on the translated amino acid sequences of the ORFs against the NCBI protein database. The mitochondrial sequences of the *L. sicilis* group were subsequently aligned with the ORFs to search for potential pseudogenes.

### Dataset construction and comparison of mitogenomes

We used only complete and circularized mitogenome sequences deposited in the NCBI GenBank until March 2024, including those of five calanoids, four harpacticoids, three siphonostomatoids and seven cyclopoids (19 species; S1 Table in S1 File). A complete mitochondrial genome of the calanoid copepod *Bestiolina similis* was recently published [46]; however, we did not find sequences deposited in NCBI (accessed on 06 Jun 2025); therefore, it was not included. Sequences deposited as ‘linear DNA’ and/or ‘partial genome’ were excluded from our study.

The evolution of the mitogenome size in Copepoda was explored using the function fastAnc in phytools v2.0 [47], which allows the reconstruction of ancestral states of a continuous variable; this analysis was performed along the Bayesian phylogeny obtained in this work (see below). We performed the analysis with RStudio 2023 v12.1.402 using the log-transformed length of the 24 complete mitogenome assemblages.

Using the qMGR program (Quantifying Mitogenome Rearrangement, [48]), an analysis of genetic rearrangement frequency was performed for each copepod species using the ancestral crustacean mitogenome proposed by Sterling-Montealegre and Prada [10] as a reference*.* Previously, we attempted to reconstruct ancestral Copepoda gene ordering using CREx [49] and TreeREx [50], but the results revealed a high level of uncertainty (S10 Fig in S1 File). Afterward, we compared the qMGR scores among copepod orders via phylogenetic analysis of variance (phylogenetic ANOVA) with those of phytools v2.0 [47] in R. Additionally, we explored the prevalence of pairs of contiguous non tRNA genes to identify conserved and derived combinations using the same reconstruction of the ancestral crustacean mitogenome. If a gene pair experienced an inversion to the opposite strand, it was considered a derived combination. The results were clustered with Euclidean distances and the UPGMA algorithm and are represented in heatmaps with the pheatmap R package v1.0.12 [51].

### Molecular evolution and selection

The concatenated alignments of the PCGs without stop codons were used to explore patterns of natural selection and identify positively selected sites in each PCG among the 24 copepods. Codon alignment was performed using the MUSCLE algorithm implemented in MEGA-X [41] for each of the 13 PCGs of the 24 copepod mitogenomes. We explored the variation in the dN/dS ratio (ω), where dN is the rate of nonsynonymous substitutions and dS is the rate of synonymous substitutions, via a maximum likelihood (ML) approach. This analysis employed Single Likelihood Ancestor Counting (SLAC) [52] through the HyPhy software package and was conducted on the Datamonkey platform (https://www.datamonkey.org/). Additionally, we conducted the analysis solely with sequences from the Calanoida Order and the Diaptomidae family, to which *Leptodiaptomus* spp. belong.

Furthermore, we estimated Ka/Ks (ω) for pairs of aligned PCG sequences using an external group for each concatenated matrix, the barnacle *Megabalanus volcano* (Thecostraca, Cirripedia) for the 24 copepods and Calanoida, and *Eurytemora affinis* for Diaptomidae, using the γ-MYN model [53] within the KaKs Calculator Toolbox 2.0 [54]. The ω ratio describes purifying selection (ω < 1), diversifying selection (ω > 1), or neutral evolution (ω = 1) acting on each PCG.

We used the branch models test with CodeML in PAML v4.9 [55] to detect whether selective pressure exists on selected branches along the phylogeny; the two-ratio model allows a background ω ratio and a different ω ratio for foreground branches of interest. In our research, the selected branches (S7 Table in S1 File) were coded as ‘1’ to represent foreground lineages, and the other species were the background branches. Likelihood ratio tests between one-ratio and two-ratio trees were conducted to estimate the significant differences in ω between selected branches and other branches. We also corrected for multiple tests with the Bonferroni method in RStudio.

### Phylogenetic analysis

A phylogenetic analysis was carried out using the concatenation of the amino acid sequences of the 13 PCGs belonging to the 24 copepod mitogenomes. The sequences were aligned using ClustalW in MEGA-X [41]. The crustacean *Megabalanus volcano* was the outgroup. The alignment is available on GitHub (https://github.com/JavierUrban/Mitogenomes_Leptodiaptomus/blob/main/data/Conca_13G_AA_CluW_BlocksTodo_PartFind.phy). Substitution models of evolution were selected with PartitionFinder2 software [56], and the models for each partition (genes: *COX 1*–*3*, *CYTB*, *ND 1*–*6* and *ATP 6*–*8*) were selected with the best Akaike Information Criterion (AIC; S9 Table in S1 File). In most cases, MTREV+I and MTZOA+I + G4 were used, which are the most common amino acid substitution models for mitochondrial genes in crustaceans [57]. Phylogenetic analyses were performed using a Bayesian inference (BI) method in MrBayes v3.2 [58], which was run with two sets of four Markov Chain Monte Carlo methods, each with 5 million initial generations, sampling once every 1,000 generations with a warmup of 25%. Chain convergence was evaluated in Tracer v1.7.2 [59]. Tree sampling was performed to generate a maximum credibility consensus tree, and the posterior probability of each branch was evaluated. Maximum likelihood (ML) phylogenetic analyses were also performed with RAxML-NG v1.0.0 [60] with 5,000 bootstrap replicates, searching for the best score. The phylogenies obtained with the two methods were visualized and edited with FigTree v1.4.4 [61].

## Results

### Assembly and annotation of five newly sequenced mitogenomes (*Leptodiaptomus* spp.)

We successfully recovered the mitochondrial sequences via two different sequencing technologies (Illumina and PacBio). This approach enabled us to obtain a minimum coverage > 20x (Table 2, S1 and S2 Figs in S1 File) for generating robust *de novo* assemblies of *L. garciai* and the population Atexcac of the *L. sicilis*-group. In the case of the Carmen, Preciosa and Quechulac populations, some inconsistencies in coverage were detected; thus, they were remapped to the mitogenome with the best quality within the *L. sicilis*-group (Atexcac).

**Fig 1:**
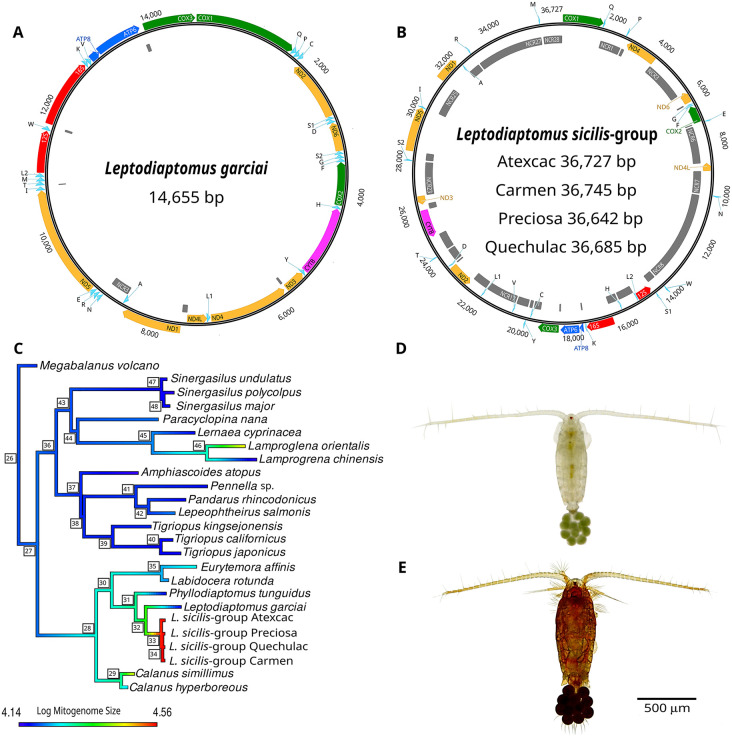
Graphic representation of the circular mitogenomes of *Leptodiaptomus* spp. **(A)** The copepod *Leptodiaptomus garciai.* **(B)** Copepods in the *Leptodiaptomus sicilis*-group. Yellow: genes from complex I (*ND* 1-6); pink: complex III (*CYTB*); green: complex IV (*COX* 1-3); red: complex V (*ATP6* and *ATP8*); dark blue: rRNAs (*12S* rRNA and *16S* rRNA); light blue: tRNAs; grey: non-coding regions (NCRs). **(C)** Evolution of the mitogenome size in Copepoda. The bar shows the correspondence between color and mitogenome size. The phylogeny is the same as that in Fig 5; the length of the branches corresponds to molecular distance. To facilitate data description, nodes are labelled 26–48; the numbers 1 - 25 correspond to the terminal branches; see the text and S6 Table in S1 File for reconstructed ancestral states of mitogenome size. **(D)** Ovigerous female of *L. garciai.* **(E)** Ovigerous female of the *L. sicilis*-group from Lake Atexcac.

All the newly assembled mitogenomes are circularized and display the general structural characteristics of metazoans and crustaceans, with 13 protein-coding genes (PCGs), 22 transfer RNA (tRNA) genes, two ribosomal RNA (rRNA) genes and several non-coding regions (NCRs) (Figs 1A and 1B, S2 and S3 Tables in S1 File). In all the annotated tRNA sequences, the corresponding anticodon was identified, and most of them adopted the characteristic cloverleaf structure, exhibiting the canonical features expected of functional tRNAs (S3 and S4 Figs in S1 File). The consistent formation of these conserved structural elements across tRNAs suggests their functionality and support their annotation as *bona fide* tRNA genes.

In all the mitogenomes, most PCGs are initiated with the codon ATN (10 in *L. garciai*, 11 in *L. sicilis-*group), with ATA as the most common form, followed by ATG and ATT (5 genes) (S2 Table in S1 File), although the start codons TTG and GTG are also present. With respect to stop codons, nine genes end with TAA, and three end with TAG; *COX*2 uses the incomplete codon TA. All the PCGs showed a strong AT bias (S2 Table in S1 File). Analysis of the relative synonymous codon usage (RSCU, S5 Fig in S1 File) revealed that there was at least one codon with a high frequency (RSCU > 1) for all the amino acids and that there were two codons (TTA for leucine and TCT for serine) whose score was > 2. There are slight differences in codon usage between the *L. garciai* and *L. sicilis-*group, with the most remarkable cases being the codons GGT (glycine) and CGT (arginine), with a high frequency only in *L. garciai* (S5 Fig in S1 File).

**Fig 2:**
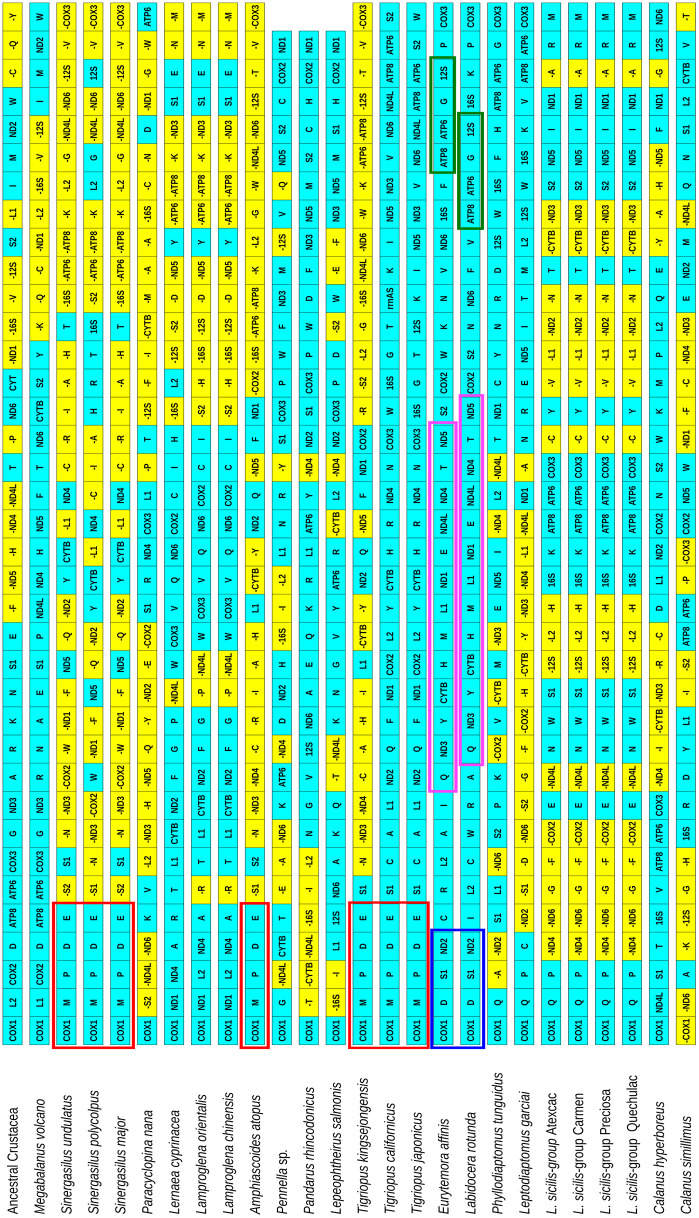
Schematic drawing of the genetic arrangements of the analysed mitogenomes. The mitogenome of the barnacle *Megabalanus volcano* and the ancestral crustacean was used as a reference (from Sterling-Montealegre and Prada [10]). Yellow and cyan indicate whether genes are encoded on the reverse or forward strands, respectively. Red boxes indicate the longest gene block shared by more than two species, all occurring in podoplean species; blue, pink and green boxes indicate the longest gene blocks shared in Calanoida. In Podoplea, several pairs of species have identical gene organization: *S. undulatus* and *S. major* and *L. orientalis* and *L. chinensis.* The four populations of *the L. sicilis*-group exhibited identical gene arrangements; thus, only one mitogenome was included in the comparative analyses (see text for details).

The gene ordering within the four members of the *L. sicilis*-group lineages is exactly the same but shows divergent features compared with *L. garciai* (Fig 2). The gene ordering of the PCGs in the latter species is almost identical to that observed in another member of the family Diaptomidae, *P. tunguidus*, although the positions of the tRNAs differ substantially (Fig 2). The nucleotide composition of the five *Leptodiaptomus* spp. mitogenomes showed an A + T bias, ranging from 68.2 to 71.0% (Table 2, S6 Fig in S1 File).

The lengths of the mitogenomes of the five *Leptodiaptomus* species vary widely because of the extension of the non-coding regions (NCRs) (Table 2; Figs. 1A and 1B; S3 Table in S1 File). The mitogenome of *L. garciai* is the smallest, with a size of 14,655 bp, including a short NCR (609 bp, 4% of the mitogenome). The mitogenomes of the copepods from the *L. sicilis-*group (Atexcac, Carmen, Preciosa, and Quechulac) are much larger, ranging from 36,642 bp to 36,745 bp, with a large NCR (22,549–22,625 bp, ≈ 62% of the mitogenome) (Table 2; Fig 1B). The nucleotide composition of the NCR is quite similar among the five *Leptodiaptomus* populations and, as the coding region, is also biased toward A + T, ranging from 72.6% (*L. sicilis-*group Atexcac) to 74.2% (*L. garciai*).

The organization and content of the NCR differed substantially between *L. garciai* and the four members of the *L. sicilis-*group. In the first species, 19 NCRs were identified, 12 with less than 10 bp, six with between 10 and 99 bp (16–89 bp), and one with 323 bp (S3 Table in S1 File, Fig 1A). In the mitogenomes of the *L. sicilis-*group, the non-coding region is divided into 31 stretches; all the stretches have the same flanking genes, and their lengths are quite similar (differing from 0 up to 143 bp), but their sequences are not identical. In the *L. sicilis*-group mitogenomes, three NCRs contain fewer than 10 bp, five with between 10 and 99 bp (43–61 bp), 16 with between 100 and 999 bp (122–952 bp), and seven with more than 1,000 bp (1,227–3,964 bp). For both species, only the NCRs > 10 bp were labelled with consecutive numbering and appear in tables and figures (S3 Table in S1 File, Fig 1B).

We identified several elements within the NCR of the *L. sicilis*-group mitogenomes, including a small region shared with *L. garciai*; this region was the only identifiable element in *L. garciai* (see below); thus, the description focused on the NCR of the *L. sicilis* species complex. The four mitogenomes share homologous tandem repeats (S4 Table in S1 File): four microsatellite-like (AT) repeats in NCRs 7, 8, 23 and 26, whereas the Atexcac and Carmen populations share two ~ 30 bp repeats in NCR23. Moreover, we identified two palindromic segments of 107 bp and 216 bp in NCR8 (S7 Fig in S1 File) in the mitogenomes of the *L. sicilis*-group.

Twenty-three open reading frames (ORFs) greater than 200 bp in length were identified, totaling 5,481 bp (24% of the NCR); in some *L. sicilis*-group populations, the ORFs are truncated because of frameshift mutations (S5 Table in S1 File). The translated amino acid sequence revealed significant similarity (e-value < 0.05) between two ORFs and two mitochondrial genes: ORF2 (total size 219 bp) in NCR6 is 62% similar to the 239 bp region of the *CYTB* gene of the calanoid copepod *Arctodiaptomus belgrati*, whereas ORF3 (264 bp) in NCR21 is 68% similar to the 267 bp region of the *COX2* gene of the crab *Echinoecus nipponicus* (S8 Fig in S1 File). No significant homologues were identified for the remaining in protein databases, suggesting potential species-specificity.

Finally, within the NCR, there are two sequences with a high degree of similarity to tRNA genes. The first is a small region of 60 bp on NCR13 of the *L. sicilis*-group and NCR6 of *L. garciai*. In both species, the sequences are located between *ATP6* and *COX3* and show 96% reciprocal similarity. BLAST analysis revealed significant similarity (e-value = 2 × 10 ⁻ ⁵) to a sequence annotated as *trnP* in *Urodontus glabratus* (Coleoptera). However, none of the predicted secondary structures exhibited the characteristic cloverleaf shape of a tRNA. Although all sequences contain the proline (NGG) anticodon, it is not positioned within a recognizable anticodon arm (S9 Fig in S1 File). The second is a sequence of 65 bp on the NCR16 of the *L. sicilis*-group, which is remarkably similar to the 71 bp sequence of *trnH* of *P. tunguidus* (e-value = 2 × 10 ⁻ ⁵; 86% similarity). The folding model produced a secondary structure with a ring and three arms, including the anticodon arm with the histidine anticodon (CAT) (not shown).

Overall, tandem repeats, palindromic segments, ORFs, and the homologous 60- and 65-bp regions together amounted to a total of 6,278 bp (672 + 5,481 + 60 + 65), representing approximately 28% of the average NCR length in the *L. sicilis* group (22,582 ± 32 bp). In the case of *L. garciai*, the homologous 60 bp region represents 10% of the NCR (609 bp).

### Comparisons across Copepoda: Evolution of mitogenome size

We recovered 19 complete mitochondrial genome assemblies from the literature and public databases, which were added to the five new assemblies described in the previous section. Thus, the analyses included 24 mitogenomes belonging to the Superorders Podoplea and Gymnoplea. In Podoplea, 14 mitogenomes were divided into three orders (Cyclopoida, Harpacticoida and Siphonostomatoida) and eight families; in contrast, Gymnoplea comprised 13 mitogenomes representing four families within its sole order, Calanoida.

With respect to the evolution of mitochondrial genome size, we inferred that the ancestral copepod mitogenome was 15,967 bp in length (node 27 in Fig 1C). No significant change was observed in the common ancestor of the Podoplea clade, which includes the orders Cyclopoida, Harpacticoida, and Siphonostomatoida (node 36: 15,528 bp), whereas an increase was predicted in the common ancestor of Gymnoplea (node 28: 18,993 bp) (Fig 1C and S6 Table in S1 File). The mitochondrial genome size subsequently decreased in podoplean species (mean for extant species = 15,626 bp; SD = 3,757 bp), except for the parasitic cyclopoid *Lamproglena orientalis*, whose genome appears to have increased in length because of complete genome duplication (unpublished; see NCBI accession OQ411235.1). In contrast, mitochondrial genome expansion continued within Gymnoplea (Calanoida; mean for extant species = 25,722 bp; SD = 10,165 bp). At the origin of the genus *Calanus* (node 29) and the family Diaptomidae (node 31), the mitogenome size reached approximately 19,000 bp and >20,000 bp, respectively. Under this scenario, both lineages exhibit size variation. In Diaptomidae, both decreases (*L. garciai* and *P. tunguidus*) and increases were observed, as well as in *Calanus* (*C. hyperboreus*). The most pronounced expansion occurred in the clade comprising the four members of the *L. sicilis*-group (node 33; 35,725 bp).

### Gene content and genetic rearrangements in Copepoda

Because the gene order was identical among the four populations of the *L. sicilis* group, we treated them as a single taxon in the gene-order analyses. Among the 21 retained mitogenomes, the gene content in Copepoda is highly conserved (13 PCGs, two rRNAs, and 22 tRNAs), with the exception of *ATP8*, which, to date, has not been reported in four podoplean species (Fig 2): *L. salmonis* [62], *P. rhincodonicus* [63], *Pennella* sp. [64] and *P. nana* [21].

Overall, we did not detect distinct patterns of gene arrangement within copepod orders, although compared with Gymnoplea, Podoplea exhibits greater internal similarity. Within Podoplea, three species pairs share identical or nearly identical gene arrangements, including tRNAs: 1) *L. chinensis* and *L. orientalis*; 2) *S. major* and *S. undulatus* are identical, and *S. polycolpus* differs from its congeners in the position of only two genes; and 3) *T. californicus* and *T. japonicus* are very similar to each other, differing only in the position of the *trnW* gene. Furthermore, several mitogenomes within Podoplea are highly similar in terms of gene order, even across different orders. For example, the gene order of *L. cyprinacea* (Cyclopoida) is highly similar to that of *Lamproglena* spp. (Cyclopoida), whereas that of *Amphiascoides atopus* (Harpacticoida) is very similar to that of *Sinergasilus* spp. (Cyclopoida). Finally, in Podoplea, seven species (three cyclopoids and four harpacticoids) share the longest identified gene block (five genes), which consists of *COX1*, *trnM, trnP, trnD*, and *trnE* (Fig 2). In contrast, within Calanoida (Gymnoplea), we did not detect significant similarities in gene order among more than two species. The pair with the greatest similarity was *Eurytemora affinis* and *Labidocera rotunda* (from different families), which shared three blocks of four genes each and one block comprising 13 genes (Fig 2).

**Fig 3:**
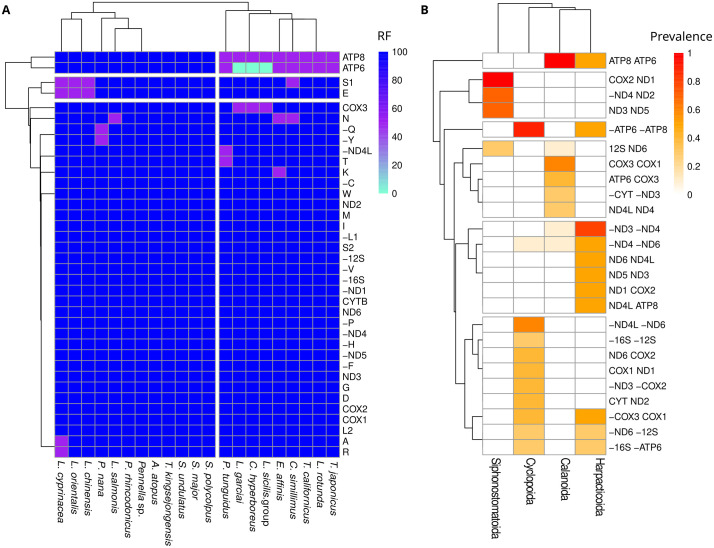
Genetic rearrangement. **(A)** Heatmap of the rearrangement frequency (RF) according to the qMGR algorithm in copepod species. For a given gene, a score of 0 (aquamarine) indicates that the two flanking genes are the same as those in the reference mitogenome; a score of 50 (purple) indicates that one flanking gene has changed, and a score of 100 (blue) indicates that both flanking genes have changed. The minus sign (-) is added when the gene appears on the reversed strand in the reference mitogenome. The reference mitogenome is the ancestral Crustacea proposed by Sterling-Montealegre and Prada [10]. **(B)** Per Order prevalence of 25 pairs of contiguous non-tRNA genes present in at least two species in 21 mitogenomes of copepods.

We then examined patterns of gene arrangement from an evolutionary perspective, i.e., the extent of change from a common ancestor, using two approaches. Reconstruction of the ancestral copepod mitogenome yielded unreliable results (S10 Fig in S1 File); therefore, we compared the 21 mitogenomes with the ancestral crustacean mitogenome proposed by Sterling-Montealegre and Prada [10]. Thus, for the first comparative approach, we applied the qMGR (Quantifying Mitogenome Rearrangement) algorithm (Fig 3A) for the 37 genes. The rearrangement frequency (RF) is high, with 24 genes showing 100% RF (i.e., both flanking genes have changed). The *ATP6* gene has an RF of 0 in three calanoids (green squares in Fig 3A) and the lowest global RF, followed by *ATP8* (average RF ± SD: 79 ± 25% and 71 ± 38%, respectively) (Fig 3A). All the species exhibited high rearrangement scores (RSs), ranging from 95% to 100%. Calanoida had the lowest RS (range 95–97%; mean ± SD: 95 ± 1%), whereas Siphonostomatoida had the highest (range 99–100%; mean ± SD: 99 ± 1%). Intermediate values were observed for Cyclopoida (95–100%; mean ± SD: 98 ± 2%) and Harpacticoida (97–100%; mean ± SD: 99 ± 2%). However, these differences were not statistically significant (phylogenetic ANOVA, *p* = 0.197).

For the second approach, we looked for pairs of non-tRNA adjacent genes. In the hypothetical ancestral crustacean mitogenome, there are six pairs (*ATP8 ATP6*; *ATP6 COX3*; *-ND4 -ND4L*; *ND6 CYTB*; *CYTB* -*ND1;* and *-ND1* -*16S*), of which only the combinations *ATP8 ATP6* and *ATP6 COX3* are observed in our dataset. The *ATP8 ATP6* pair is present in the forward strand in all calanoids plus two harpacticoids (43% of copepods), but in eight podopleans, it underwent a translocation to the reverse strand (38% of species) and was considered a different block (Fig 3B); the *ATP6 COX3* gene pair was also observed in the ancestral forward strand but only in three calanoids (14% of species). Therefore, the *ATP8 ATP6 COX3* block, which is very common in Arthropoda, appears only in three calanoid species (14%). Finally, the ancestral pair -*ND4*-*ND4L* is observed only in the putatively “original” metazoan version, ND4L ND4, in two calanoids.

On the other hand, we identified 45 novel combinations of adjacent non-tRNA genes, that is, combinations not present in the ancestral crustacean genome, 22 of which occurred in only one species. Overall, there are 25 gene pairs (conserved and novel) present in at least two species, and we used them to look for similarities (clusters) among orders. Seventeen of these pairs are exclusive to a given order (four in Calanoida, six in Cyclopoida, four in Harpacticoida, and three in Siphonostomatoida; Fig 3B). Harpacticoida and Cyclopoida shared the greatest number of gene pairs (five), but Harpacticoida was grouped with Calanoida (Fig 3B) because of the medium/high frequency of their shared pairs. This results in Siphonostomatoida being the most divergent, with three exclusive gene pairs of high frequency.

### Selection analysis

**Fig 4:**
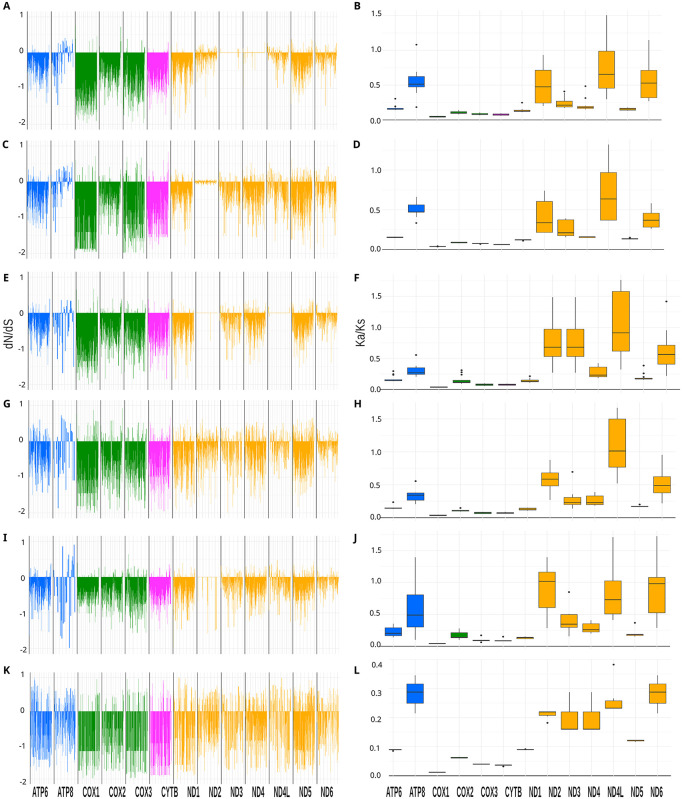
Selection Analysis. Selection signatures for each codon of the 13 PCGs (dN/dS); in the left column, the SLAC method is used, and in the right column, the Ka/Ks ratio is calculated for the PCGs of mitochondrial genomes. **(A)** and **(B)**, Within the Class Copepoda; **(C)** and **(D)**, Superorder Gymnoplea (order Calanoida); **(E)** and **(F)**, Superorder Podoplea; **(G)** and **(H)**, Order Cyclopoida; **(I)** and **(J)**, Orders Harpacticoida + Siphonostomatoida; **(K)** and **(L)**, Family Diaptomidae. Blue is Complex V (*ATP*), green is Complex IV (*COX*), pink is Complex III (*CYTB*) and yellow is Complex I (*ND*).

Codon-level selection analyses of the 13 PCGs using SLAC [52] revealed more sites (codons) under purifying selection than under positive selection, with a variable proportion evolving neutrally (Fig 4; S7 Table in S1 File). The genes of Complex IV (*COX*s) experienced the greatest proportion of sites under purifying selection (Copepoda average = 71 ± 9%), especially *COX1* (81%), which was well ahead of *CYTB* (67%), and the lowest proportion was observed in complex V (*ATP*s) (Copepoda average = 16 ± 2%). Sites under positive selection are present in only three genes of Complex I: *ND2* (Copepoda, 1 site; Gymnoplea, 30 sites), *ND4* (Copepoda, 1 site) and *ND4L* (Podoplea, 1 site). We did not find signatures of positive selection in Cyclopoida, Harpacticoida+Siphonostomatoida (H + S) or Diaptomidae.

A complementary approach to detect signatures of selection was to estimate pairwise Ka/Ks ratios. Across all the clades, the lowest Ka/Ks values (suggesting strong purifying selection) are observed for the CYTB and Complex IV genes (COXs), followed by three genes from Complex I (*ND1*, *ND4*, and *ND5*) and *ATP6*. The Ka/Ks ratios of four Complex I genes exceed 1 in some groups, potentially reflecting positive selection: *ND2* in Podoplea and H + S, *ND3* in Podoplea, *ND4L* in all groups except Diaptomidae, and *ND6* in H + S. The Ka/Ks ratio is also greater than 1 for the *ATP8* gene in H + S because of the parasitic species *L. orientalis*. No signatures of positive selection were detected within the family Diaptomidae.

We subsequently applied the branch-site model [65] to assess whether specific sites experienced positive selection along particular branches (lineages). We evaluated branches corresponding to the same groups as those in the other analyses, except for Copepoda (S8 Table in S1 File). Overall, eight genes across all the taxonomic groups suggest positive selection. Four genes from complex I (*ND1*, *ND2*, *ND4*, and *ND5*) show signatures of selection in all the evaluated branches, except for Gymnoplea. The *CYTB* has a high probability of positive selection only in the deepest nodes (Gymnoplea and Podoplea). Within Complex IV, *COX3* shows evidence of positive selection in Gymnoplea, Cyclopoida, and H + S, whereas *COX1* does so only in Podoplea. In Complex V, *ATP6* is under positive selection in the Gymnoplea, Podoplea, and H + S branches.

### Phylogenetic analysis

**Fig 5:**
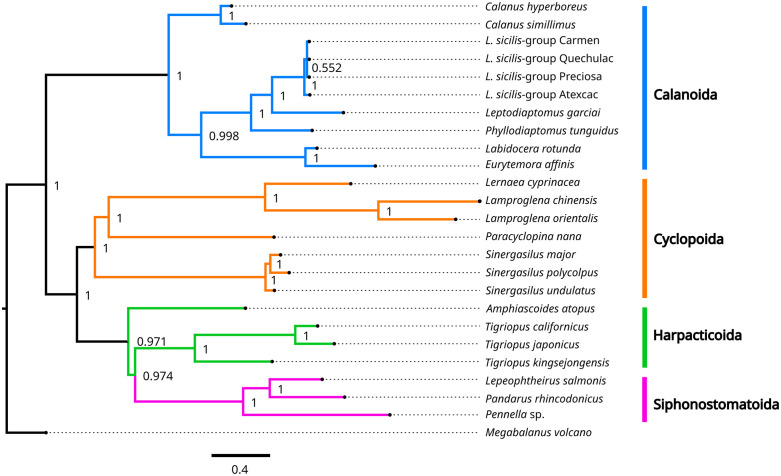
Phylogenetic reconstruction. Phylogenetic tree with a concatenation of the 13 protein-coding genes from 24 copepods using a Bayesian inference method. The numbers beside the nodes are the posterior probabilities, and the scale bar represents 0.4 estimated substitutions per site.

The phylogenetic analyses conducted with BI (Fig 5) and ML (S11 Fig in S1 File) provided trees with similar, but not identical, topologies. At first glance, the most notable difference concerns the placement of *Amphiascoides atopus*, which appears within Harpacticoida in the ML phylogeny but as a sister taxon to Harpacticoida + Siphonostomatoida in the Bayesian reconstruction. However, bootstrap support at the deeper nodes of the ML phylogeny within the Podoplea clade is low (42–67%), reducing confidence in this topology. In contrast, the BI phylogeny shows high posterior probabilities and was therefore used for downstream analyses. Aside from the placement of *A. atopus*, the BI phylogeny recovers the four copepod orders sampled in this study as monophyletic groups; within Podoplea, Harpacticoida and Siphonostomatoida are recovered as sister clades.

The family Diaptomidae was recovered as a strongly supported monophyletic group in both phylogenies. *L. garciai* is clearly differentiated, whereas relationships among the four lineages of the *L. sicilis*-group remain poorly resolved. In particular, one node with a very low posterior probability (0.552) forms a polytomy involving the Carmen, Quechulac, and Preciosa populations.

## Discussion

### Mitochondrial genome assembly and characteristics

Various factors can hinder the amplification and assembly of mitochondrial genomes, including genomic rearrangements, base composition biases, regions with high repeat content, and stable secondary structures that interfere with polymerase progression [21,66], often resulting in fragmented mitogenomes [67]. In this study, despite the *L. sicilis*-group copepod mitogenomes exhibiting these features, the use of a hybrid assembly strategy (combining PacBio and Illumina sequencing) allowed us to obtain a complete, circularized *de novo* mitogenome from the Atexcac population. Short-read sequences from the other *L. sicilis*-group populations (Carmen, Preciosa, and Quechulac) were subsequently remapped to obtain complete *de novo* mitogenomes. For *L. garciai*, *de novo* assembly was successfully achieved using only short-read sequences, possibly because of its genomic architecture, which displayed the typical features of metazoan mitogenomes.

In general, *Leptodiaptomus* copepod mitogenomes exhibited an A + T bias (~60%), which is consistent with the pattern observed in the other copepod mitogenomes analysed here. Within the PCGs, the A + T composition varied across genes but remained within the 60–70% range, which is typical of crustaceans [23]. Additional features associated with elevated A + T content were also present, including NCRs composed of an average composition of ~73% A + T. Pronounced A + T bias has been proposed to decrease structural stability, generating unstable non-coding sequences, and is thereby associated with a higher frequency of genetic rearrangements [24]. This instability may represent an important evolutionary force, particularly in copepods, which will be further explored below. In addition, A + T bias can shape mitochondrial function by altering key regulatory regions and favouring the preferential use of AT-rich codons, potentially driving lineage-specific evolutionary trajectories [68]. The most frequent start codon was ATA, whereas TAA was the most frequent stop codon. Moreover, most AT-rich codons presented RSCU values > 1. Codon usage in PCGs plays an important role in regulating gene expression and may be shaped by translational selection [69]. In the *COX2* gene, the stop codon is incomplete (TA) and is resolved through post-transcriptional polyadenylation, a common feature in crustaceans [70]. While we addressed several of these implications, such as genomic rearrangements, further integration of transcriptional analyses will be essential to clarify how these patterns in *Leptodiaptomus* copepods are related to transcription activity and, ultimately, to their capacity to adapt to ecologically contrasting environments.

### Evolution of Mitogenome Size: Variability of the NCR

The era when animal mitogenomes were considered compact, with adjacent genes and short non-coding intergenic regions (NCRs) [71], is now behind us. While this concept still applies to some copepod species, such as the harpacticoid *Amphiascoides atopus* (NCR: 154 bp), four of the *de novo* mitogenomes presented here (*L. sicilis*-group) exhibit the opposite pattern: their mitochondrial size exceeds 36,000 bp, of which 62% corresponds to the NCR. Furthermore, the mitogenomes of the four *L. sicilis*-group members are the largest known to date in copepods. Previously, the largest complete assembly in Copepoda belonged to the parasitic cyclopoid *Lamproglena orientalis* (28,462 bp, unpublished; see [72,73]). However, the origin of the expansion was entirely different: in *L. orientalis,* it was attributed to a complete duplication of the 37 genes, whereas in the *L. sicilis*-group, it was due to NCR enlargement. The other copepod species with large NCRs belong to the genus *Calanus: C. glacialis, C. simillimus* and *C. finmarchichus* (with mitogenome lengths of 27,342 bp, 27,876 bp and 29,462 bp, respectively) [20]. Thus, the NCR length is highly variable within Copepoda, ranging from <2% of the mitogenome in the harpacticoid *A. atopus* to 62% in the *L. sicilis*-group reported here. For comparison, the largest animal mitogenome published to date belongs to the parasitic cnidarian *Enteromyxum leei*, measuring 187,936 bp, 98.5% of which corresponds to the NCR (including ORFs) [9].

When the NCR content in *L. sicilis*-group mitogenomes was examined, we identified several structural elements, including palindromes, tandem repeats, and ORFs. Inversions and ORF duplications have been reported to play key roles in mitogenome expansion [74,75], although in the *L. sicilis*-group, ORFs contributed only 24% of the NCR. Whereas tandem repeats have been reported in mitochondrial NCRs of copepods [19,20], the finding of 23 ORFs is remarkable, as no ORFs of a significant length have been found in other copepods with large NCRs, such as *C. glacialis* or *C. finmarchicus* [20]. Two ORFs showed similarities to the mitochondrial genes *CYTB* and *COX2* of *Leptodiaptomus* spp., probably originated by gene duplications [76]. ORFs in non-coding regions may result from nuclear genetic insertions, horizontal transfer, or mitochondrial gene duplications and strand displacements, subsequently leading to loss of functionality, as observed in other metazoan mitogenomes [76,77]. Although specific functions cannot yet be assigned to these elements, studies of animal mitochondrial genomes suggest that NCRs can contain signals involved in replication, transcriptional regulation, transcript processing, and repeat-mediated genome rearrangements [8,13,14]. Nevertheless, non-coding expansions may also reflect neutral accumulation or weak selective constraint, and their functional relevance may differ markedly among lineages. The coexistence of palindromes, tandem repeats, ORFs, and tRNA-like sequences within the NCR of the *L. sicilis*-group should not be interpreted as evidence of function; rather, it points to a set of candidate regulatory or structural features whose potential roles in copepod mitogenome maintenance, organization, or expression remain to be experimentally validated.

Additionally, we found regions similar to the *trnP* and *trnH* sequences. In metazoan mitochondrial genomes, these features generally arise through a multifaceted process involving gene duplication, subsequent degeneration, and transposition events [13], along with identity shifts driven by anticodon mutations, a phenomenon commonly referred to as gene remodelling or tRNA gene recruitment [78,79]. However, improved annotation of these genomic regions is necessary to better understand their evolutionary impact on copepod mitogenomes, as their function and complex evolutionary dynamics suggest that they may play important roles in adaptation to new environmental conditions [80]. Although our findings are insufficient to fully explain the expansion of *L. sicilis*-group copepod mitogenomes, the proportion of identified elements is greater than that reported for other copepods. In other calanoids and arthropods, NCRs are typically characterized by complex repeats lacking sequence similarity among closely related species, suggesting limited homology and/or high mutation rates [8,20].

In Copepoda, the length of the coding region shows limited variability (≤401 bp); thus, reconstructions of the ancestral states of mitogenome size essentially reflect NCR evolution. Our results suggest that early copepods possessed mitogenomes comparable in size to those of ancestral arthropods (15,000–16,000 bp; [7]). Two contrasting trends subsequently emerged: a generally conserved but slightly reduced genome size in Podoplea and an expansion in the lineage leading to Calanoida. Notably, Diaptomidae, which is the most diverse calanoid family in continental waters [81], includes lineages with both the greatest reductions and the greatest mitogenome expansions, even the largest known mitogenome in Copepoda. Given our limited knowledge of NCR structure and function, it is not yet possible to attribute an adaptive role to its expansion in calanoids. Increasing the mitogenome dataset, particularly within Diaptomidae, will thus be essential for elucidating the evolutionary mechanisms underlying both NCR reduction and rapid expansion in this clade.

### Gene content and rearrangements: Most and least conserved features in mitogenomes

One of the most common features of metazoan mitogenomes is the conserved number and type of genes they retain [82]. Copepoda is no exception, as most species preserve a canonical set of 37 genes: 13 protein-coding genes (PCGs), two ribosomal RNA genes (12S and 16S rRNA), and 22 transfer RNAs. However, among the mitogenomes published as “complete”, four cases stand out (one cyclopoid species and three siphonostomatoids), in which the *ATP8* gene sequence was either reported as absent [21,63,64] or mentioned in the publication but not properly deposited or annotated [62]. *ATP8* has highly divergent sequences and extremely variable lengths, often creating annotation difficulties [83]. There are previous cases in which some authors concluded that this gene was absent, for example, in molluscs [84], but careful manual annotation or additional sequencing later confirmed its presence [83]. In copepods, *ATP8* is the shortest mitochondrial PCG, ranging from 85 to 165 bp, and is likely overlooked. Interestingly, the reported absence of *ATP8* appears to be restricted to Podoplean species, suggesting that its loss or extreme modification may have a phylogenetic component, as observed in flatworms [85] and mussels [83]. For our analyses, we retained the published gene sets, despite the absence of *ATP8*, to avoid further limiting the scarce collection of circularized mitogenomes. However, expanding and validating copepod mitogenome databases with new sequencing and annotation technologies will be important to minimize overinterpretation.

We could not find distinctive patterns in gene organization either in Copepoda as a class or in any of its constituent orders. Among extant podoplean species, we found a block of five adjacent genes shared by 50% of the podoplean species, but its prevalence is not high enough to be considered a distinctive gene block of the superorder. This lack of structural patterns is consistent with the extremely high rates of gene rearrangements we found, which are the highest among arthropods, excluding hexapods [10]. In contrast, other closely related crustacean groups, such as branchiopods [24], exhibit largely conserved arrangements of PCGs and tRNAs, retaining most features of pancrustacean mitogenome models, with variation mainly restricted to tRNA positions. The sharp contrast in the rearrangement rates of Branchiopoda (23%; [10]) and Copepoda (>95% in this study) is particularly noteworthy, given their similarly long evolutionary histories and successful colonization of both marine and freshwater environments [15,22].

When we compared the PCG pairs present in the extant copepod species to the reconstruction of ancestral crustacean gene organization [10], we found 45 new combinations, half of which were present in only one species, suggesting the increased occurrence of reshuffling events in copepod mitogenomes. On the other hand, of the six ancestral gene pairs, only two are conserved and have a reduced prevalence: *ATP8 ATP6* and *ATP6 COX3*. Both gene pairs, along with their combination *ATP8 ATP6 COX3*, are the most prevalent blocks in Chordata, Arthropoda and Echinodermata (>95%; [82]). Conversely, in Copepoda, *ATP8 ATP6* is present in only 43% of species, whereas the translocated version, *-ATP6 -ATP8*, with a prevalence of 0.5% in Arthropoda, is present in 38% of species.

Notably, the –*ND4* –*ND4L* pair, present in the ancestral crustacean organization and 98% of extant arthropods, is absent in copepods, whereas the putatively ancestral metazoan *ND4L ND4* version [82] is present in two calanoids. Like *ATP8 ATP6*, the adjacency of the *ND4L ND4* pair is attributed to the partial overlap of their sequences [86], and its prevalence in metazoans is considered evidence that this gene pair is under strong purifying selection [82]. Thus, the disruption of this block in 95% of copepods may represent a significant event in the evolutionary history of the group.

Overall, our results indicate that genetic rearrangement is a widespread mitogenomic pattern in copepods, lacking clear, universal rearrangement “hotspots” across the group. These rearrangements may be driven by various mechanisms, including tandem duplication–random loss (TDRL) [87], illegitimate recombination of repeated sequences [88], and tRNAs acting as mobilizing elements within the mitochondrial genome. However, we cannot determine whether any of these mechanisms alone could explain the high rearrangement rate, as the limited number of complete genomes available and the contrasting lineages represented do not provide sufficient resolution.

### Signatures of adaptive and neutral evolution

We examined selection footprints by the ratio of synonymous to nonsynonymous mutations (Ka/Ks and dN/dS ratios) and performed a branch-site (BS) analysis to detect genes evolving under positive selection along multiple phylogenetic branches.

Considering the entire subclass Copepoda, we identified a suite of highly conserved genes with low Ka/Ks ratios and a high proportion of codons under strong purifying selection (dN/dS ratios). This group includes *ND1* (Complex I), *CYTB* (Complex III), and Complex IV genes (*COX1*, *COX2*, and *COX3*). *COX1* is often reported as the gene under the strongest purifying selection, not only in copepods [72] but also in other aquatic animals, including penguins [89] and sea turtles [90]. *ND1* also emerged as a highly conserved gene, as noted in penguins [91], but was overlooked in earlier copepod studies. While other Complex I genes show signals of positive selection (see below), *ND1* evolution may be constrained by metabolic demands in copepods and other aquatic animals.

Nevertheless, dN/dS ratios and branch-site analysis revealed that, with the exceptions of *ATP8* and *ND6*, all the mitochondrial genes presented evidence of positive selection. Considering the contrasting ecological conditions in which copepods have diversified, these findings may point to differential electron transport efficiency in oxidative phosphorylation, potentially shaped by variations in energy demand [89,92]. Comparable patterns of positive selection have been observed in other invertebrates, including crustaceans, and are linked to differences in temperature, habitat depth, light, and oxygen conditions [93].

Finally, in the *ATP8* gene, most sites are likely evolving under neutral conditions, and a small number of sites are under purifying selection. Thus, as observed in other animal groups, a considerable portion of its encoded protein is highly variable and, in some cases, is recognized only by its conserved secondary structure [82].

Our findings therefore support the view that mitochondrial protein-coding genes can evolve adaptively to optimize function in copepods; however, additional studies, particularly differential expression analyses, are needed to identify the environmental drivers of this process.

### Phylogenetic relationships

Reconstructing phylogenetic relationships within Copepoda has proven challenging, even when diverse systematic characteristics and advanced analytical approaches are used. In this study, on the basis of 13 concatenated PCGs, we inferred two phylogenetic hypotheses using the ML and BI methods, with the latter yielding higher support values. These hypotheses differed in terms of the phylogenetic placement of *A. atopus.* In the BI tree, *A. atopus* emerged as a sister clade to Harpacticoida + Siphonostomatoida with a high posterior probability. Within this phylogeny, Siphonostomatoida and Harpacticoida were recovered as sister clades, a relationship consistent with previous phylogenetic hypotheses based on morphological characteristics [94] or mitochondrial genes [19]. However, alternative topologies have been reported, placing Siphonostomatoida as a sister group to Cyclopoida [16,72], whereas other studies have recovered Cyclopoida as a sister group to Harpacticoida [95]. Clearly, elucidating the sequence of ancient branching events that gave rise to the three podoplean clades (Harpacticoida, Siphonostomatoida, and Cyclopoida) remains a formidable challenge, perhaps because of the severely limited number of complete mitogenomes currently available. Given the ecological and economic importance of Copepoda, expanding its molecular resources is imperative for advancing our understanding of its evolutionary history.

For Cyclopoida and Calanoida, both phylogenies recovered identical topologies, with congruent placements and nested relationships, in agreement with previous studies [16,19]. In contrast, relationships within the *L. sicilis* group remained unresolved, as most internal nodes received low statistical support. This limited resolution is consistent with a scenario of recent divergence and may also reflect insufficient phylogenetic signal in the mitochondrial markers, potentially associated with purifying selection.

## Conclusion

Several decades ago, the mitogenome was considered relatively conserved from an evolutionary perspective, given the importance of its function and its strong dependence on the nuclear genome. Currently, it is clear that mitogenomes are highly dynamic and exhibit a complex and heterogeneous evolutionary landscape both within genomes and across lineages. In the present study, we show that Copepoda provides a clear example of this dynamism. The only feature that is consistently conserved across most copepod species is the metazoan set of 37 genes (13 PCGs, two ribosomal genes, and 22 tRNAs). In contrast, both the genome size and the rate of gene rearrangement have evolved markedly.

In this study, we report the largest known copepod mitogenome in populations of the *Leptodiaptomus sicilis*-group. This finding is not an isolated case but rather reflects an evolutionary trend observed in Calanoida. This clade has undergone substantial expansions in the length of its non-coding regions. In contrast, the more recently diverged Podoplean clade shows little change in noncoding region length. Another striking feature of copepod mitogenomes is their high rate of gene rearrangement, which is among the highest reported in arthropods (excluding Hexapoda). This process has erased most ancestral gene blocks that remain conserved in other invertebrates and has generated numerous novel combinations, making it difficult to identify distinctive gene blocks even at the order level. It is therefore premature to draw conclusions about the causes and consequences of these patterns, as the roles of much of the NCR and of gene order in copepod fitness remain unknown.

The number of complete copepod mitogenomes remains limited and biased, with data available for only four of the seven copepod orders, which restricts our ability to fully understand their evolution. Nevertheless, we hope our findings provide a solid foundation and stimulate further research to improve our understanding of the molecular mechanisms underlying the adaptation of copepods to past and future environmental challenges.

## Supporting information

S1 File S1 Table. Species used in the comparative analyses and NCBI accession numbers of their mitogenomes. The barnacle *Megabalanus volcano* (Crustacea) was the outgroup. **S2 Table.** Size, start and stop codons and composition of the 13 PCGs of the copepods in *L. garciai* and *L. sicilis*-group. **S3 Table.** Position and size of the non-coding regions (NCR) of the copepods in the *L. sicilis*-group and *L. garciai.* Only sequences > 10 bp are included. **S4 Table.** Repetitive regions identified in the mitogenomes of the copepods *Leptodiaptomus sicilis*-group. **S5 Table.** ORFs > 200 bp present in the mitogenomes of the *L. sicilis*-group. In gray are ORFs that are truncated due to frameshift mutations. **S6 Table.** Reconstructed ancestral states in the evolution of mitogenome size (MtSize) in Copepoda are displayed in the phylogeny of Fig 1 in the main text. The numbers 1–25 correspond to the terminal branches (24 copepod species plus the barnacle *Megabalanus volcano*). **S7 Table.** Number of sites (codons) subject to selection for the 13 PCGs using the SLAC method with *P* = 0.05; H + S = Siphonostomatoida + Harpacticoida, (+) = Positive selection, (-) Purifying selection, %N = Percentage of neutral sites. **S8 Table.** Estimation ΔLRT of nested *codeml* branch-site model A (*p* < 0.05). Only genes with statistically significant results for positive selection are shown. **S9 Table.** Number of partitions per gene and evolutionary models selected in Partition Finder 2 for Bayesian Inference (BI) and Model-Test for Maximum Likelihood (ML) analyses. **S1 Fig.** Estimated coverage of the mitochondrial genome assemblies of the copepods *Leptodiaptomus sicilis*-group. At the top, the coverage of the mitochondrial genome assembly of the *L. sicilis*-group Atexcac is shown using long-read sequences (PacBio), followed by the coverage of short-read sequences (Illumina) of the four populations. The bottom panel displays the genetic arrangement for the four populations. **S2 Fig.** Estimated coverage of the mitochondrial genome assemblies of the copepod *Leptodiaptomus garciai* using short-read sequences (Illumina). The bottom panel displays the genetic arrangement. **S3 Fig.** Schematic representation of the secondary structure of transfer RNA (tRNA) genes identified in the mitogenomes of the four populations of the *L. sicilis*-group. **S4 Fig.** Schematic representation of the secondary structure of transfer RNA (tRNA) genes identified in the mitogenomes of the four populations of the copepod *L. garciai.* **S5 Fig.** Relative synonymous codon usage (RSCU) of the mitochondrial protein-coding genes of the *Leptodiaptomus* copepods analysed in this study. Capital letters on top of each chart correspond to the one-letter code for the 20 amino acids. **S6 Fig.** Percentage of nucleotide composition of 24 mitochondrial genomes of copepods. **S7 Fig.** Secondary structure and sequences of two palindromes found in the non-coding region of the mitochondrial genome of the *L. sicilis*-group. **S8 Fig.** Sequence alignments of *L. sicilis*-group mitochondrial NCR6 vs. CYTB and NCR21 vs. COX2. **S9 Fig.** Sequence alignment and secondary structure of NCR13 from the *L*. *sicilis*-group and NCR6 from *L*. *garciai* vs. tRNA-*Pro* of *Urodontus glabratus* (Coleoptera). Blue circle: 5’ end; red circle: 3’ end. **S10 Fig.** Ancestral gene order reconstruction of Copepoda. The colour at each node represents the probability of consistency: green = consistent, yellow = k-consistent, and red = inconsistent (reversal). The letters on the branches indicate the type of rearrangement: inversion (I), translocation (T), deletion or duplication (TDLR). **S11 Fig.** Phylogenetic reconstruction of a concatenation of 13 mitochondrial genes from 24 copepod species using Maximum Likelihood. Bootstrap values are shown at each node.
(ZIP)
