# Supplementary material for: The Copepoda mitogenome as a dynamic evolutionary landscape
Source: PLoS One. 2026 Jun 10;21(6):e0350115. doi: 10.1371/journal.pone.0350115 (PMC13252756; doi:10.1371/journal.pone.0350115)
Supplement: S1 File — Species used in the comparative analyses and NCBI accession numbers of their mitogenomes. The barnacle Megabalanus volcano (Crustacea) was the outgroup. S2 Table. Size, start and stop codons and composition of the 13 PCGs of the copepods in L. garciai and L. sicilis-group. S3 Table. Position and size of the non-coding regions (NCR) of the copepods in the L. sicilis-group and L. garciai. Only sequences > 10 bp are included. S4 Table. Repetitive regions identified in the mitogenomes of the copepods Leptodiaptomus sicilis-group. S5 Table. ORFs > 200 bp present in the mitogenomes of the L. sicilis-group. In gray are ORFs that are truncated due to frameshift mutations. S6 Table. Reconstructed ancestral states in the evolution of mitogenome size (MtSize) in Copepoda are displayed in the phylogeny of Fig 1 in the main text. The numbers 1–25 correspond to the terminal branches (24 copepod species plus the barnacle Megabalanus volcano). S7 Table. Number of sites (codons) subject to selection for the 13 PCGs using the SLAC method with P = 0.05; H + S = Siphonostomatoida + Harpacticoida, (+) = Positive selection, (-) Purifying selection, %N = Percentage of neutral sites. S8 Table. Estimation ΔLRT of nested codeml branch-site model A (p < 0.05). Only genes with statistically significant results for positive selection are shown. S9 Table. Number of partitions per gene and evolutionary models selected in Partition Finder 2 for Bayesian Inference (BI) and Model-Test for Maximum Likelihood (ML) analyses. S1 Fig. Estimated coverage of the mitochondrial genome assemblies of the copepods Leptodiaptomus sicilis-group. At the top, the coverage of the mitochondrial genome assembly of the L. sicilis-group Atexcac is shown using long-read sequences (PacBio), followed by the coverage of short-read sequences (Illumina) of the four populations. The bottom panel displays the genetic arrangement for the four populations. S2 Fig. Estimated coverage of the mitochondrial genome assemblies of the c [file pone.0350115.s001.zip › S8 Table.docx]

**Supplementary Information**

**S8 Table.** Estimation ΔLRT of nested *codeml* branch-site model A (*p* <0.05). Only genes with statistically significant results for positive selection are shown.

| **Branch** | **Gene** | **lnL (null)** | **lnL (alternative)** | **ΔLRT** | ***P*-value** | **p-value (Bonferroni)** |
| --- | --- | --- | --- | --- | --- | --- |
| Gymnoplea (Calanoida) | COX3  CYTB  ATP6 | -12255.197  -15924.311  -11174.126 | -12245.058  -15919.805  -11169.509 | 20.278  9.0129  9.235 | 6.70E-06  0.0026  0.0023 | 8.70E-05  0.0348  0.0308 |
| Podoplea | COX1  CYTB  ND2  ATP6 | -18864.817  -15939.828  -15328.357  -11179.799 | -18860.905  -15935.746  -15321.434  -11175.350 | 7.823  8.164  13.845  8.897 | 0.0051  0.0042  0.0002  0.0028 | 0.0167  0.0167  0.0025  0.0167 |
| Cyclopoida | COX3  ND1  ND3  ND4  ND5 | -12265.788  -15241.763  -6217.5659  -24220.215  -31011.852 | -12260.6461  -15235.958  -6211.906  -24215.699  -31004.498 | 10.284  11.610  11.319  9.031  14.707 | 0.0013  0.0006  0.0007  0.0026  0.0001 | 0.0160  0.0078  0.0092  0.0318  0.0015 |
| Harpacticoida + Siphonostomatoida | COX3  ND1  ND4  ATP6 | -12260.227  -15234.718  -24221.128  -11171.980 | -12255.923  -15230.494  -24211.283  -11168.108 | 8.608  8.448  19.690  7.744 | 0.0033  0.0036  9.10E-06  0.0053 | 0.0401  0.0438  0.0001  0.0646 |
| Diaptomidae | ND1  ND2  ND4  ND5 | -15240.613  -15331.401  -24219.497  -31011.837 | -15234.228  -15325.731  -24214.933  -31006.838 | 12.770  11.339  9.128  9.999 | 0.0003  0.0007  0.0025  0.0015 | 0.0042  0.0091  0.0301  0.0187 |
